# Sulfonamide Inhibition Studies of a New β-Carbonic Anhydrase from the Pathogenic Protozoan *Entamoeba histolytica*

**DOI:** 10.3390/ijms19123946

**Published:** 2018-12-08

**Authors:** Silvia Bua, Susanna Haapanen, Marianne Kuuslahti, Seppo Parkkila, Claudiu T. Supuran

**Affiliations:** 1 Sezione di Scienze Farmaceutiche e Nutraceutiche, Neurofarba Dept., Università degli Studi di Firenze, Via U. Schiff 6, 50019 Sesto Fiorentino, Florence, Italy; silvia.bua@unifi.it; 2 Faculty of Medicine and Health Technology, Tampere University, 33100 Tampere, Finland; Haapanen.Susanna.E@student.uta.fi (S.H.); Marianne.Kuuslahti@staff.uta.fi (M.K.); seppo.parkkila@staff.uta.fi (S.P.); 3 Fimlab Ltd., Tampere University Hospital, 33100 Tampere, Finland

**Keywords:** carbonic anhydrase, metalloenzymes, protozoan, *Entamoeba histolytica*, sulfonamides, sulfamates, inhibitor

## Abstract

A newly described β-carbonic anhydrase (CA, EC 4.2.1.1) from the pathogenic protozoan *Entamoeba histolytica*, EhiCA, was recently shown to possess a significant catalytic activity for the physiologic CO_2_ hydration reaction (k_cat_ of 6.7 × 10^5^ s^−1^ and a k_cat_/K_m_ of 8.9 × 10^7^ M^−1^ s^−1^). A panel of sulfonamides and one sulfamate, some of which are clinically used drugs, were investigated for their inhibitory properties against EhiCA. The best inhibitors detected in the study were 4-hydroxymethyl/ethyl-benzenesulfonamide (K_I_s of 36–89 nM), whereas some sulfanilyl-sulfonamides showed activities in the range of 285–331 nM. Acetazolamide, methazolamide, ethoxzolamide, and dichlorophenamide were less effective inhibitors (K_I_s of 509–845 nM) compared to other sulfonamides investigated here. As β-CAs are not present in vertebrates, the present study may be useful for detecting lead compounds for the design of more effective inhibitors with potential to develop anti-infectives with alternative mechanisms of action.

## 1. Introduction

The pathogenic protozoan *Entamoeba histolytica* is the leading cause of diarrhea globally, producing a disease called amebiasis. Endemic in poor communities in developing countries, amebiasis emerged as an important infection among travelers returning from such countries as well as immigrants residing in the developed world [1,2,3]. The invasive forms of the *E. histolytica* infection may include liver cyst formation, which can produce complications such as pleural effusion due to the rupture of the cysts [4,5,6]. Rarely, the cysts may disseminate to other extra-intestinal organs, such as the brain or pericardium, with fatal consequences. Amebiasis causes around 70,000 deaths annually and is the third cause of death due to parasites worldwide [7,8,9]. The pharmacological treatment relies on the use of metronidazole and related compounds (e.g., tinidazole), which show multiple adverse side effects, being rather toxic, mutagenic and carcinogenic, and led to the emergence of resistance [4,9]. Unfortunately, better therapeutic alternatives are lacking, and the nitroimidazoles do not effectively eradicate the luminal cysts of the parasite life cycle. Therefore, it has become necessary to administer a luminal agent, such as nitazoxanide or the aminoglycoside paromomycin, which are expensive new drugs, which is difficult to use in developing countries [4,9]. Ultimately, the gold(I) derivative Auranofin, used for the treatment of rheumatoid arthritis, has entered clinical drug development as an antiparasitic agent targeting amebiasis [4,5,6,7,8,9]. However, the treatment options are few, their effectiveness is not very high, and the presently available drugs have many side effects and led to the development of drug resistance. All these facts make the search for new anti-amoeba targets of great relevance [4,5,6,7,8,9,10].

In recent years, we have investigated the role of the metalloenzymes, carbonic anhydrases (CAs, EC 4.2.1.1), in various pathogenic organisms belonging to the bacteria, fungal or protozoan domains [11,12,13]. These enzymes effectively catalyze the reaction between CO_2_ and water, with the formation of bicarbonate (HCO_3_^−^) and protons (H^+^), being among the very fast catalysts known in nature [14,15,16,17,18,19,20]. CAs are multifunctional enzymes which play central roles in various physiological, biochemical, and metabolic processes, such as acid-base homeostasis, respiratory gas exchange, electrolytes secretion, biosynthesis of urea, glucose, fatty acids, and carbamoyl phosphate, and also in the ionic transport, muscular contraction (in vertebrates), and photosynthesis (in plants and algae). Seven distinct genetic families. i.e., the α, β, γ, δ, ζ, η, and θ class CAs are known to date, with a wide distribution in organisms all over the tree of life [21,22,23,24,25,26,27]. The CA classes do not share any significant sequence and structural identity since they are a paradigmatic example of convergent evolution at the molecular level [11,12,13]. Recently, we have shown that the inhibition of the α- or β-CAs from the pathogenic protozoans *Trypanosoma cruzi* [28] or *Leishmania* spp. [29] has a potent anti-protozoan effect, with the possibility to inhibit the growth of the pathogen. Considering that the genome of *E. histolytica* has been published [30], we decided to investigate in detail whether the β-CA present in this pathogenic protozoan may have a similar role to the enzymes investigated earlier in other pathogenic protozoans [28,29]. Here we report an investigation of the catalytic activity and the sulfonamide/sulfamate inhibition profile of the recombinant enzyme belonging to the β-class, identified in the genome of the pathogenic protozoan *E. histolytica*, denominated EhiCA.

## 2. Results and Discussion

We produced the β-CA of *E. histolytica,* EhiCA, in the *E. coli* expression system (see Experimental for details) and obtained 21/25 kDa doublet polypeptide and additional polypeptides of about 50 and 75 kDa detected by SDS-PAGE. These four polypeptide bands were subjected to mass spectrometric identification, which showed that they all represent EhiCA. This result suggests that EhiCA, similar to other β-CAs [31], can exist as dimers and higher oligomerization forms [32,33,34].

The catalytic activity of the recombinant EhiCA (for the CO_2_ hydration reaction), has been measured by using a stopped-flow technique [35], comparing its kinetic parameters with those of other such enzymes, belonging to the α- (e.g., hCA I and II, where h stays for human isoform) or β-class CAs (e.g., mtCA 1 and mtCA 2 from the pathogenic bacterium *Mycobacterium tuberculosis* [31,32]). Data in Table 1 show that EhiCA has a significant catalytic activity (for the physiologic CO_2_ hydration reaction), with a k_cat_ of 6.7 × 10^5^ s^−1^ and a k_cat_/K_m_ of 8.9 × 10^7^ M^−1^ s^−1^, being, thus, 1.8 times more effective as a catalyst compared to the slow human isoform hCA I (considering the k_cat_/K_m_ values). Furthermore, like most enzymes belonging to the CA superfamily, EhiCA was inhibited by acetazolamide (**AZA**, 5-acetamido-1,3,4-thiadiazole-2-sulfonamide), a standard, clinically used sulfonamide CA inhibitor [1,2,3]. Thus, EhiCA shows a catalytic activity similar to that of mtCA 2 and hCA I, being a highly effective catalyst for the hydration of CO_2_, whereas its inhibition by acetazolamide is similar to the behavior of mtCA 1, which has a low affinity for this inhibitor, with a K_I_ of 480 nM, comparable to that of EhiCA, of 509 nM (Table 1).

**Figure 1:**
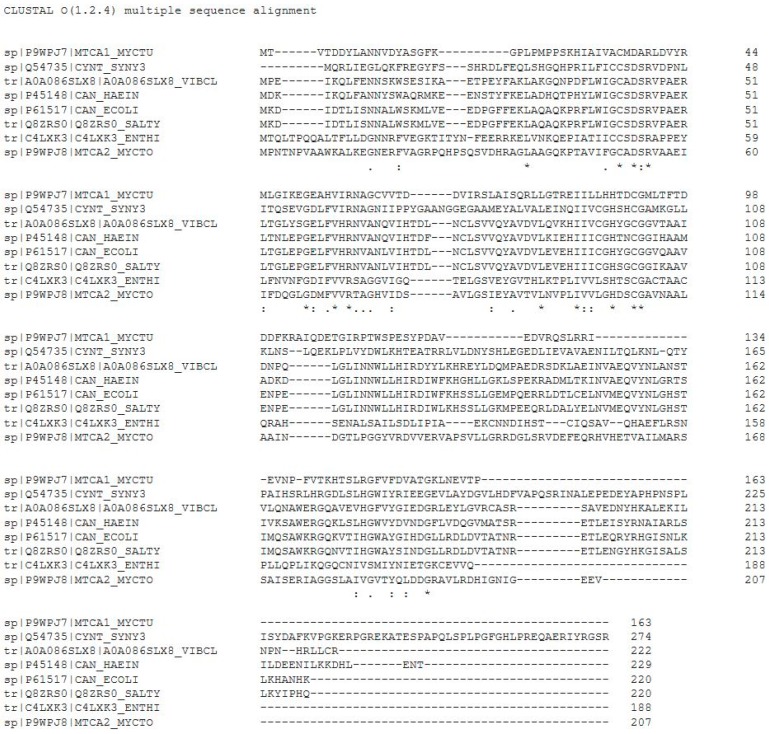
Multi-alignment of the amino acid sequences of the β-CAs from *M. tuberculosis* (isoform MTCA1_MYCTU), *Synechocystis* sp. (SYNY3), *V. cholerae* (VIBCL), *H. influenzae* (HAEIN), *E. coli* (ECOLI), S. *typhimurium* (SALTY), *E. histolytica* (ENTHI), and *M. tuberculosis* (isoform MTCA2_MYCTO) [21,30,31,32,33,34,35,36]. Conserved amino acids depicted by an asterisk (*), semiconserved ones by (.) or (:).

To rationalize the effective catalytic activity of EhiCA, we aligned the amino acid sequence of this protein with that of other β-CAs, such as those from the pathogenic bacteria *Haemophilus influenza* [21], *Vibrio cholera* [33], *Escherichia coli* [21], *Salmonella typhimurium* [36], two isoforms from *Mycobacterium tuberculosis* [31,32], and the cyanobacterium *Synechocystis* sp. PCC 6803 [34] (Figure 1).

As seen from data in Figure 1, similar to all β-CAs investigated to date, EhiCA has the conserved three zinc(II) ligands, Cys50, His103, and Cys106 (the fourth ligand is presumably a water molecule/hydroxide ion) as well as the catalytic dyad constituted by the pair Asp52-Arg54 (conserved in all enzymes belonging to this class) [21,31,32,33,34,36], which contributes to the enhancement of the nucleophilicity of the water coordinated to the metal ion. The presence of these conserved amino acids and all the structural elements connected to them may explain the good catalytic activity of EhiCA reported in this paper (Table 1), although the X-ray crystal structure of this enzyme is not yet resolved.

**Figure 2:**
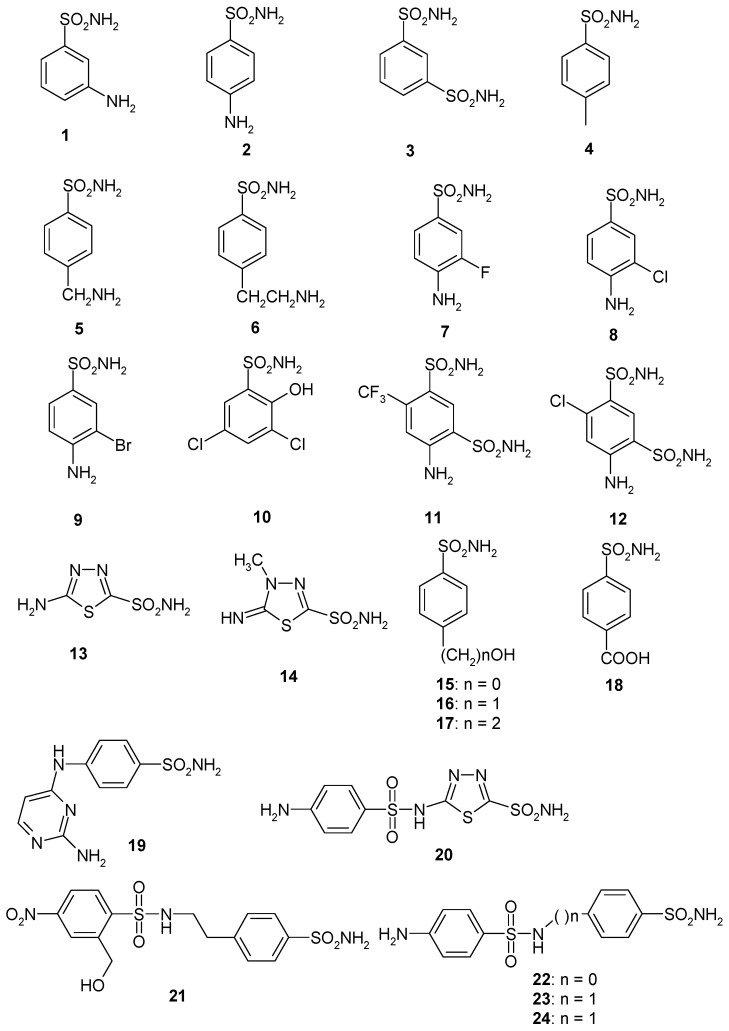

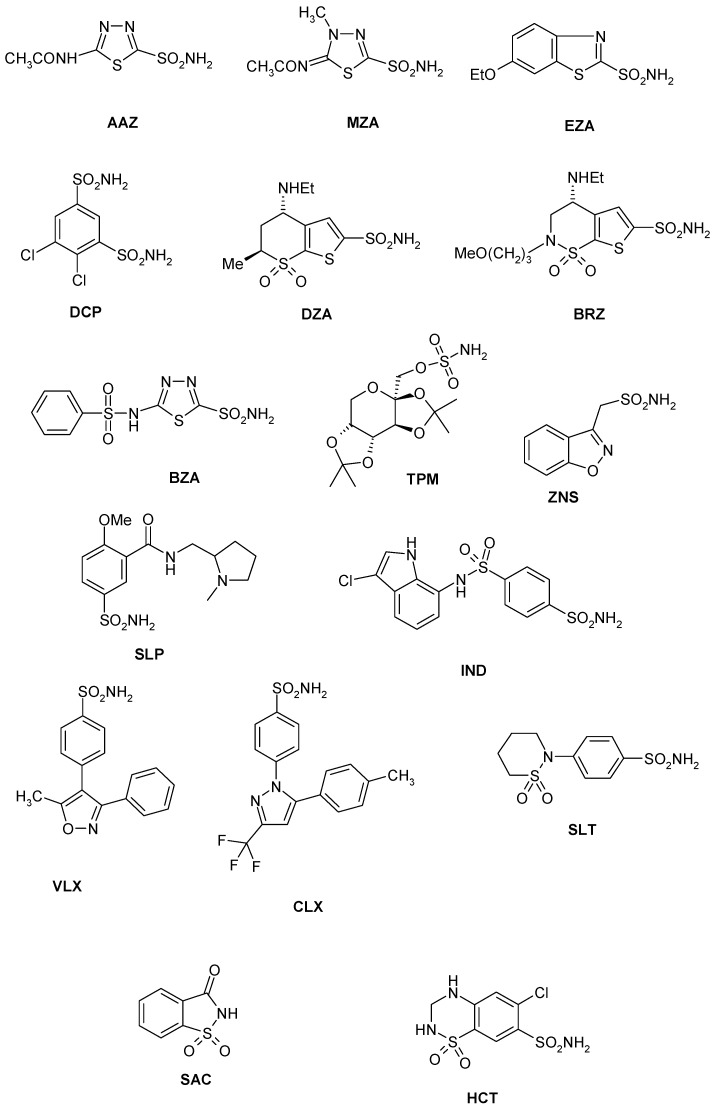
Sulfonamide (**1–24**) and sulfonamide/sulfamate derivatives **(AAZ–HCT)** investigated as *Entamoeba histolytica* (EhiCA) inhibitors in the present study.

Considering that the sulfonamides are the main class of CA inhibitors (CAIs) [11,12,13], we investigated the inhibition of EhiCA with a panel of such derivatives, some of which are clinically used drugs like diuretics, antiglaucoma, antiepileptics, antiobesity or antitumor agents [37,38,39,40] (Figure 2 and Table 2). The structures of the sulphonamides/sulfamates included in our study are shown in Figure 2. They include acetazolamide **AAZ**, methazolamide **MZA**, ethoxzolamide **EZA** and dichlorophenamide **DCP** (the classical, systemically acting antiglaucoma CA inhibitors) [11,12], dorzolamide **DZA** and brinzolamide **BRZ**, topically-acting antiglaucoma drugs, benzolamide **BZA,** topiramate **TPM**, zonisamide **ZNS,** and sulthiame **SLT** [11,12,13,37,38,39,40]. Sulpiride **SLP**, indisulam **IND,** celecoxib **CLX,** and valdecoxib **VLX**, as well as saccharin and the diuretic hydrochlorothiazide **HCT** were also included in the assay [11,12,13]. The simpler sulfonamides **1**–**24** are known to possess CA inhibitory properties against many mammalian and prokaryotic such enzymes [25] and are also the building blocks for obtaining more complex CAIs [41,42,43].

The following structure-activity relationship (SAR) can be drawn from the data of Table 2:

(i) The most effective EhiCA inhibitors were the two simple compounds **16** and **17**, 4-hydroxymethyl/ethyl-benzenesulfonamides, which showed K_I_s ranging between 36 and 89 nM, with the longer linker derivative (**17**) being a more effective CAI compared to the hydroxymethyl one **16**. It should also be noted that **17** is a weaker hCA II inhibitor (K_I_ of 125 nM) and a quite ineffective hCA I inhibitor (K_I_ of 21 µM), making it a slightly ameba-CA—selective compound.
(ii) Several sulfonamides were slightly less effective as EhiCA inhibitors, with K_I_s ranging between 285 and 521 nM. They include **18**–**24** and acetazolamide **AAZ** (Table 2). Apart from **18** (4-carboxy-benzenesulfonamide) and **19** (a pyrimidinylamino-benzenesulfonamide), the remaining derivatives **20**–**24** belong to the sulfanilyl-sulfonamide class of CAIs, which possess an elongated molecule, shown to interact favorably with many other CAs belonging to the β-class [15,20,21] and, thus, leading to effective inhibitors. For the homologous series of **22**–**24**, the efficacy as EhiCA inhibitors increases with the increase of the linker between the two aromatic rings. **AAZ** and **20** contain the 1,3,4-thiadiazole-2-sulfonamide motif present in many potent CAIs. In this case, aminobenzolamide **20** is a more effective EhiCA inhibitor compared to **AAZ**. It is interesting to note that **BZA**, lacking the amino moiety present in **20**, but with an identical scaffold, is a very weak CAI, with a K_I_ of 2471 nM (whereas it is a very potent hCA I and II inhibitor). Thus, minor structural changes in the molecule of the inhibitor lead to drastic effects on their inhibitory profiles against various CAs, including the one form the parasitic protozoan investigated here.
(iii) The following compounds showed modest EhiCA inhibitory properties: **3**–**6**, **11**, **13**–**15**, **MZA**, **EZA**, **DCP**, and **IND**, with K_I_s ranging between 567 and 951 nM. They belong to heterogeneous classes of sulfonamides, most of them being benzenesulfonamides (apart **13** and **14** which are the deacetylated precursors of **AAZ** and **MZA**, thus, heterocyclic derivatives). A special mention regards **15**, which is structurally related to the most effective EhiCA inhibitors detected here, compounds **16** and **17**. Indeed, **15** is 9–20 times a weaker EhiCA inhibitor compared to **16** and **17**, although they differ only by one or two CH_2_ functionalities. From these data, it is again obvious that SAR is very sensitive to small changes in the molecule of the inhibitor and that the 4-hydroxyalkyl-substituted-benzenesulfonamides may lead to highly effective and isoform-selective CAIs targeting the enzyme from this parasite.
(iv) Weak, micromolar inhibition against EhiCA was observed with **1**, **2**, **10**, **12**, **DZA**, **BRZ**, **BZA**, **TPM**, **ZNZ**, **SLT**, and **HCT** (K_I_s ranging between 1.91–9.59 µM) as discussed earlier. In addition, these derivatives belong to heterogeneous classes of derivatives, but overall one may observe that they possess a bulkier scaffold and more substituents on the aromatic/heterocyclic ring compared to the effective EhiCA inhibitors described above.
(v) The ineffective compounds as EhiCA inhibitors (K_I_ > 10 µM) detected here were **7**–**9** (halogenated sulfanilamide derivatives), sulpiride **SLP**, the COX-2 inhibitors **CLX** and **VLX** (possessing a bulky, Y-shaped molecule), and saccharin **SAC**, the only acylated, secondary sulfonamide included in the study.
(vi) The inhibition profile of EhiCA with sulfonamides/sulfamates is very different from those of the human isoforms hCA I and II, but only two compounds, **16** and **17** showed selectivity for the protozoan over the human isoforms (Table 2).

## 3. Experimental

### 3.1. Vector Construction

We produced the EhiCA as a recombinant protein in *E. coli*. The DNA sequence was retrieved from UniProt and modified for recombinant protein production and purification to include N-terminal polyhistidine tag. We provided the sequence of the insert, and the actual construction of the plasmid vector was performed by GeneArt (Invitrogen, Regensburg, Germany). The structure of the insert was specifically modified for production in *E. coli*. The insert was ligated into a modified plasmid vector, pBVboost [44].

### 3.2. Production of the Protein

The freeze-dried plasmid was prepared according to the manufacturer’s manual. Deep-frozen BL21 Star^TM^ (DE3) cells (Invitrogen, Carlsbad, CA, USA) were slowly melted on ice. 25 µL of the melted cell suspension and 1 µL of the plasmid solution were combined. The suspension was kept on ice for 30 min. Then the heat shock was performed by submerging the suspension containing tube into +42 °C water for 30 s and after that, incubated on ice for 2 min. 125 µL of S.O.C. Medium (Invitrogen, Carlsbad, CA, USA) was added to the tube, and the tube was incubated for 1 h with constant shaking (200 rpm) at +37 °C. Growth plates (gentamycin-LB medium ratio 1:1000) were prewarmed at +37 °C for 40 min. Twenty microliters and 50 µL of the suspension were spread on two plates, which were incubated overnight at +37 °C. A volume of 5 mL preculture was prepared by inoculating single colonies from growth plates to LB medium with gentamycin (ratio 1:1000). It was then incubated overnight at +37 °C with constant shaking of 200 rpm. Then the production was executed according to pO-stat fed bacth protocol, which is essentially as described in Määttä et al. [45]. There were some alterations to the previously described protocol: The fermentation medium did not contain glycerol as the cell line used did not require it. The induction of the culture was performed with 1 mM IPTG 12 h after starting the fermentation. The temperature was decreased to 25 °C at the time of the induction. Culturing was stopped after 12 h of the induction with the OD 34 (A_600_). The cells were collected by centrifugation, and the wet weight of cell pellet was 303 g. The fermentation was performed by Tampere facility of Protein Services (PS). The cell pellet (approximately 35 g) was suspended in 150 mL of binding buffer containing 50 mM Na_2_HPO_4_, 0.5 M NaCl, 50 mM imidazole, and 10% glycerol (pH 8.0) and the suspension was homogenized with EmulsiFlex-C3 (AVESTIN, Ottawa, ON, Canada) homogenizer. The lysate was centrifuged at 13,000× *g* for 15 min at 4 °C, and the clear supernatant was mixed with HisPur™ Ni-NTA Resin (Thermo Fisher Scientific, Waltham, MA, USA) and incubated for 2 h at room temperature on a magnetic stirrer. Then, the resin was washed with the binding buffer and collected onto an empty column with EMD Millipore™ vacuum filtering flask (Merck, Kenilworth, NJ, USA) and a filter paper. The protein was eluted from the resin with 50 mM Na_2_HPO_4_, 0.5 M NaCl, 350 mM imidazole and 10% glycerol (pH 7.0). The protein was re-purified with TALON^®^ Superflow™ cobalt resin (GE Healthcare, Chicago, IL, USA). The eluted protein fractions were diluted to binding buffer (50 mM Na_2_HPO_4_, 0.5 M NaCl, and 10% glycerol pH 8.0) so that the imidazole concentration was under 10 mM. The protein binding and elution were performed as described above. The purity of the protein was determined with gel electrophoresis (SDS-PAGE) and visualized with PageBlue Protein staining solution (Thermo Fisher Scientific, Waltham, MA, USA). Mass spectrometric identification of the obtained polypeptide bands was performed in the Tampere University Facility of Protein Services. Protein fractions were pooled and concentrated with 10 kDa Vivaspin^®^ Turbo 15 centrifugal concentrators (Sartorius™, Göttingen, Germany) at 4000× *g* at 4 °C. Buffer exchange in 50 mM TRIS (pH 7.5) was done with the same centrifugal concentrators. His-tag was cleaved from the purified protein by Thrombin CleanCleave Kit (Sigma-Aldrich, Saint Louis, MO, USA) according to manufacturer’s manual.

### 3.3. CA Activity and Inhibition Measurements

An Sx.18Mv-R Applied Photophysics (Oxford, UK) stopped-flow instrument has been used to assay the catalytic activity of various CA isozymes for CO_2_ hydration reaction [35]. Phenol red (at a concentration of 0.2 mM) was used as indicator, working at the absorbance maximum of 557 nm, with 10 mM HEPES (pH 7.5, for α-CAs) or TRIS (pH 8.3, for β-CAs) as buffers, 0.1 M NaClO_4_ (for maintaining constant ionic strength), following the CA-catalyzed CO_2_ hydration reaction for a period of 10 s at 25 °C. The CO_2_ concentrations ranged from 1.7 to 17 mM for the determination of the kinetic parameters and inhibition constants. For each inhibitor at least six traces of the initial 5–10% of the reaction have been used for determining the initial velocity. The uncatalyzed rates were determined in the same manner and subtracted from the total observed rates. Stock solutions of inhibitors (0.1 mM) were prepared in distilled-deionized water, and dilutions up to 1 nM were done thereafter with the assay buffer. Enzyme and inhibitor solutions were pre-incubated together for 15 min before assay, to allow for the formation of the enzyme–inhibitor complex. The inhibition constants were obtained by non-linear least-squares methods using PRISM 3 and the Cheng–Prusoff equation, as reported earlier [46,47,48].

## 4. Conclusions

In the search for alternative drug targets for anti-protozoan agents, we report the first sulphonamide/sulfamate inhibition study of EhiCA, a β-class CA from the parasitic protozoan *Entamoeba histolytica*. The strong enzyme inhibitors identified in the study were 4-hydroxymethyl/ethyl-benzenesulfonamide (K_I_s of 36–89 nM), which were also selective for inhibiting the protozoan over the human CA isoforms. Some sulfanilyl-sulfonamides also showed good activities, with inhibition constants in the range of 285–331 nM. Acetazolamide, methazolamide, ethoxzolamide and dichlorophenamide, clinically used agents, were less effective EhiCA inhibitors (K_I_s of 509–845 nM) compared to other sulfonamides investigated here. As β-CAs are not present in vertebrates, the present study may be useful for detecting lead compounds for the design of more effective inhibitors with the potential to develop anti-infectives with alternative mechanisms of action. Compounds, such as the strong enzyme inhibitors detected here, 4-hydroxymethyl/ethyl-benzenesulfonamide, may also be used as pharmacologic tools for understanding the role played by this enzyme in the life cycle of the protozoan.

## Figures and Tables

**Table 1:** Kinetic parameters for the CO_2_ hydration reaction catalyzed by the human cytosolic isozymes hCA I and II (α-class CAs) at 20 °C and pH 7.5 in 10 mM HEPES buffer and 20 mM Na_2_SO_4_, and the β-CA from M. tuberculosis (mtCA 1 and 2) and form *E. histolytica* EhiCA, measured at 20 °C, pH 8.3 in 20 mM TRIS buffer and 20 mM NaClO_4_. Inhibition data with the clinically used sulfonamide acetazolamide (5-acetamido-1,3,4-thiadiazole-2-sulfonamide) are also provided [35].

Enzyme	Activity Level	Class	k_cat_	k_cat_/K_m_	K_I_ (Acetazolamide)	Ref
			(s^−1^)	(M^−1^ s^−1^)	(nM)	
hCA I	moderate	α	2.0 × 10^5^	5.0 × 10^7^	250	[12]
hCA II	very high	α	1.4 × 10^6^	1.5 × 10^8^	12	[12]
mtCA 1	moderate	β	3.9 × 10^5^	3.7 × 10^7^	480	[32]
mtCA 2	high	β	9.6 × 10^5^	9.3 × 10^7^	9.8	[32]
EhiCA	high	β	6.7 × 10^5^	8.9 × 10^7^	509	this work

**Table 2:** Inhibition of the human isoforms hCA I and hCA II, and *Entamoeba histolytica* (EhiCA) from *Entamoeba histolytica* with sulfonamides **1**–**24** and the clinically used drugs **AAZ–HCT**, by a stopped-flow, CO_2_ hydrase assay [35].

Inhibitor/Enzyme Class	K_I_ * (nM)		
	hCA I ^a^	hCA II ^a^	EhiCA
	α	α	β
**1**	28,000	300	2363
**2**	25,000	240	6011
**3**	79	8	951
**4**	78,500	320	833
**5**	25,000	170	567
**6**	21,000	160	798
**7**	8300	60	>10,000
**8**	9800	110	>10,000
**9**	6500	40	>10,000
**10**	7300	54	4656
**11**	5800	63	742
**12**	8400	75	1911
**13**	8600	60	821
**14**	9300	19	579
**15**	5500	80	772
**16**	9500	94	89
**17**	21,000	125	36
**18**	164	46	383
**19**	109	33	521
**20**	6	2	385
**21**	69	11	368
**22**	164	46	331
**23**	109	33	290
**24**	95	30	285
**AAZ**	250	12	509
**MZA**	50	14	845
**EZA**	25	8	746
**DCP**	1200	38	790
**DZA**	50,000	9	6444
**BRZ**	45,000	3	3051
**BZA**	15	9	2471
**TPM**	250	10	3100
**ZNS**	56	35	9595
**SLP**	1200	40	>10,000
**IND**	31	15	822
**VLX**	54,000	43	>10,000
**CLX**	50,000	21	>10,000
**SLT**	374	9	6727
**SAC**	18,540	5959	>10,000
**HCT**	328	290	3402

* Errors in the range of 5–10% of the reported data, from 3 different assays (data not shown). ^a^ Human recombinant isozymes, stopped flow CO_2_ hydrase assay method, from References [11,12,13,14,15].
